# Supplementary material for: The claustrum enhances neural variability by modulating the responsiveness of the prefrontal cortex
Source: Nat Commun. 2025 Nov 28;16:10767. doi: 10.1038/s41467-025-65809-6 (PMC12663370; doi:10.1038/s41467-025-65809-6)
Supplement: Supplementary file 1 — Supplementary Information [file 41467_2025_65809_MOESM1_ESM.pdf]

Supplementary Figures

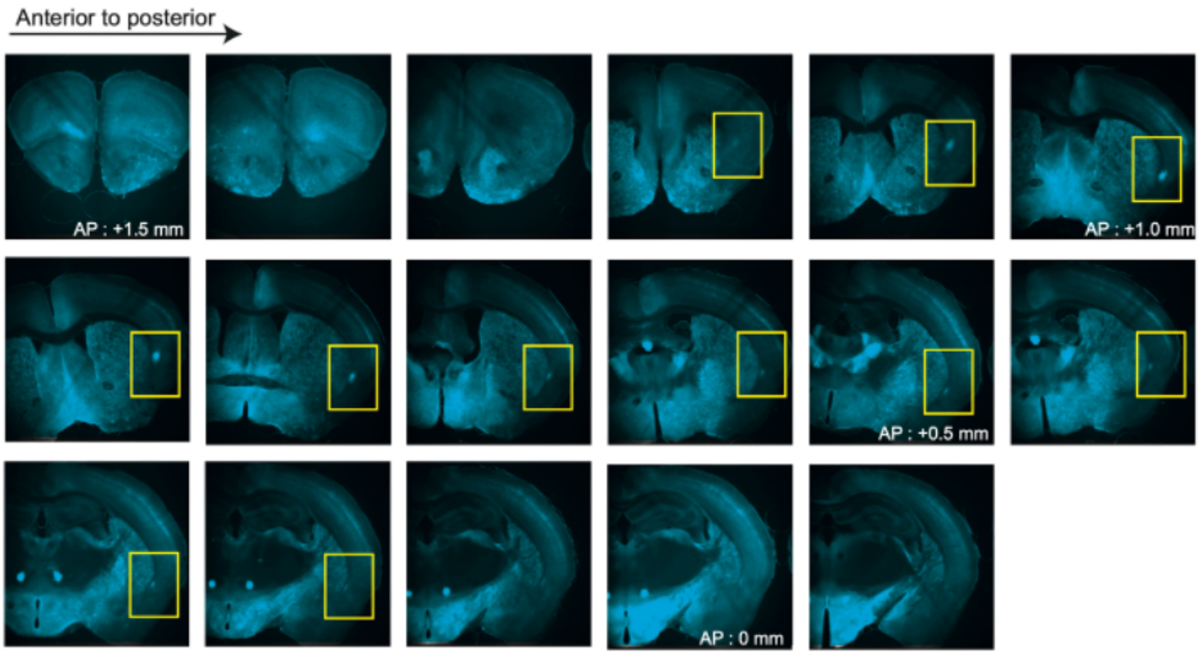

Supplementary Figure 1: Coronal brain sections illustrating the distribution of claustrum cell labeling across anterior-posterior sections of the claustrum.

The images are organized sequentially from anterior to posterior, with the first image on the left representing the most anterior section and the last image on the right representing the most posterior section. The sections start at approximately 1.5 mm anterior to the bregma, and claustrum labeling is evident from 1.2 mm, if not before, to 0.2 mm anterior to the bregma, covering nearly 1mm of the claustrum. Yellow boxes indicate the location of the claustrum.

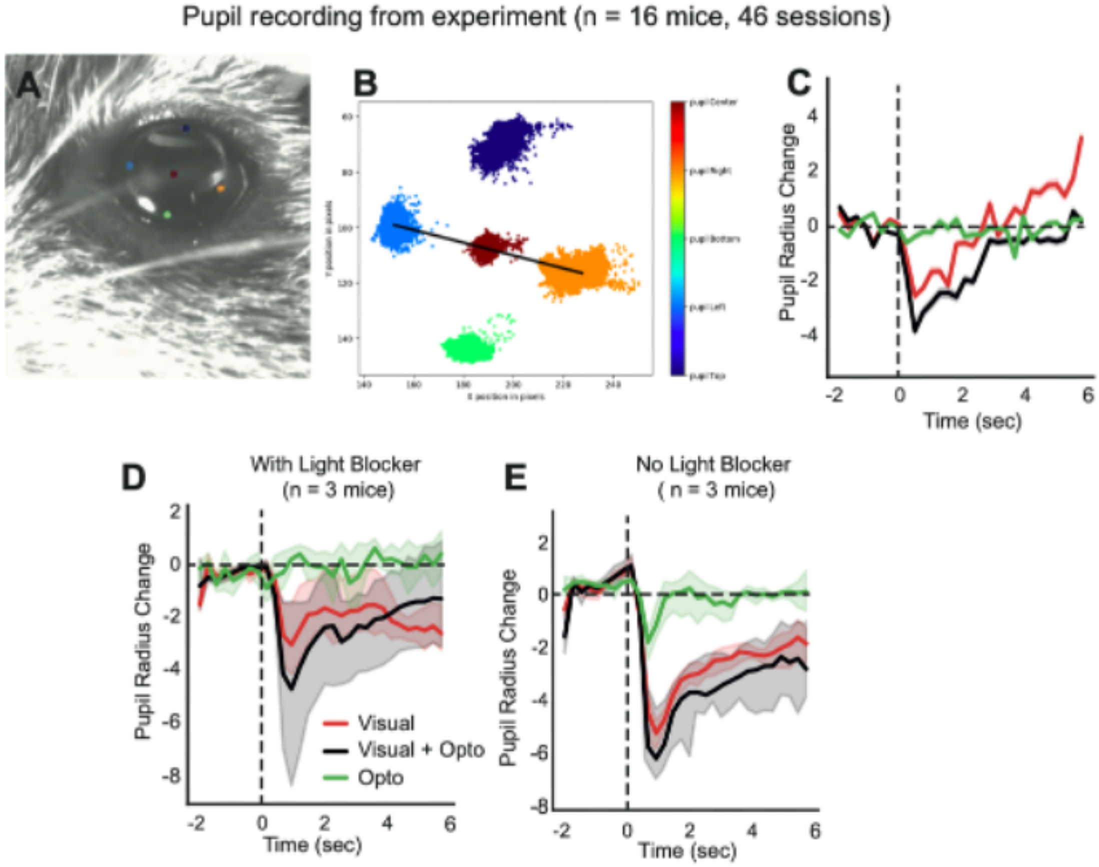

Supplementary Figure 2: Control experiments demonstrating efficacy of light blocking during optogenetic stimulation.

(A) Example infrared image of the eye during pupil tracking. Colored dots indicate keypoints annotated and tracked using a custom-trained DeepLabCut model (pupil center, top, bottom, left, and right). (B) Representative output from DeepLabCut showing the spatial distribution of tracked keypoints used to compute pupil radius across time. (C) Average pupil radius change (relative to baseline) across 16 mice (46 sessions) during visual (red), optogenetic (green), and combined (black) trials. (D) Pupil responses recorded in a dedicated control experiment (3 mice) using a custom light blocker around the objective. Optogenetic stimulation alone did not evoke pupil constriction, confirming the absence of visual contamination. (E) In the absence of a light blocker (same stimulation parameters), optogenetic stimulation evoked mild pupil constriction, indicating that stimulation light can only activate the visual system less than visual stimulation. Shaded regions indicate SEM across mice.

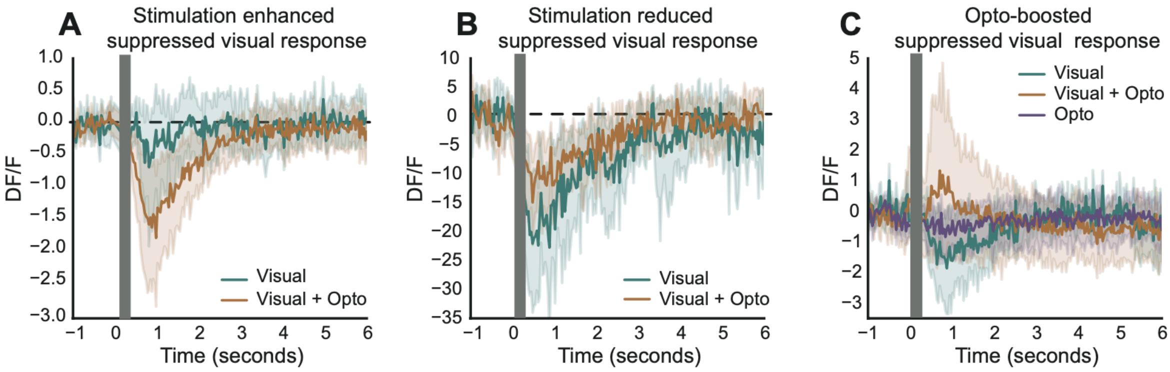

Supplementary Figure 3: Various neural modulation with the claustrum axon activation through photo-stimulation

The time course of neuronal calcium responses (ΔF/F) illustrates the modulatory effects of the claustrum axon photostimulation on dPFC neuron responses to visual stimuli. Individual plots show different modulatory effects on suppressed visual responses when combined with optogenetic stimulation.

Supplementary Table 1: Summary statistics of neuronal responsiveness across experimental conditions

The mean, minimum, maximum, and standard deviation of the percentage of neurons classified as responsive across animals, grouped by different cell responsiveness conditions. Sensory refers to cells significantly responsive to visual stimuli; Opto to optogenetic stimulation; and SensoryOpto to cells significantly responsive when visual and optogenetic stimuli are presented simultaneously. Values reflect animal-level averages.

Cell Responsiveness Categories	Mean %	Min %	Max %	Std %	Excited Mean %	Inhibited Mean %
Sensory	19.592	8.795	28.118	5.311	48.052	51.948
Opto	29.061	19.545	40.508	7.275	33.037	66.963
SensoryOpto Only (Opto-boosted)	13.193	10.436	17.241	2.178	43.930	56.070
**Convergent Categories**						
Sensory + Opto	4.409	2.889	7.585	1.398	32.205	67.795
Opto + SensoryOpto	7.319	3.822	12.385	2.641	36.289	63.711
Sensory + Opto + SensoryOpto	4.806	2.020	8.028	2.086	42.861	57.139
Sensory + SensoryOpto	5.033	3.013	8.754	1.467	43.954	56.046

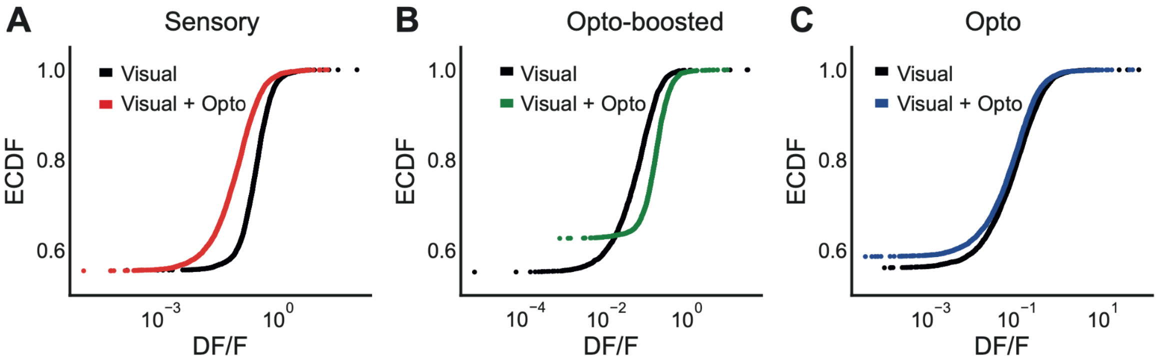

Supplementary Figure 4: **Empirical cumulative distribution function (ECDF) analysis of neural responses to combined visual stimuli presentation and axon activation through photo-stimulation**

A) The ECDF plot shows the neural responses under different conditions. The visual + optogenetic stimulation (Visual + Opto) curve (red) is consistently above the visual-only curve (black) across all points, indicating broader neuronal engagement when visual stimuli are combined with optogenetic activation. B) The ECDF curves for opto-boosted neurons reveal a more rapid increase in the visual + optogenetic condition, suggesting that more neurons quickly reached the response threshold with optogenetic activation. The steeper rise of the Visual + Opto curve (green) implies that neurons achieve the specified response level more swiftly, highlighting enhanced neural activity at the collective level for opto-boosted neurons. C) The ECDF plot for the optogenetic-only condition (blue) shows the response in the presence of optogenetic stimulation alone compared to visual-only stimulation (black). ECDF plots show the central 90% of the distribution (5th–95th percentile range) to focus on the bulk of the data and minimize the influence of outliers. As a result, the curves begin near y = 0.05 rather than at 0.

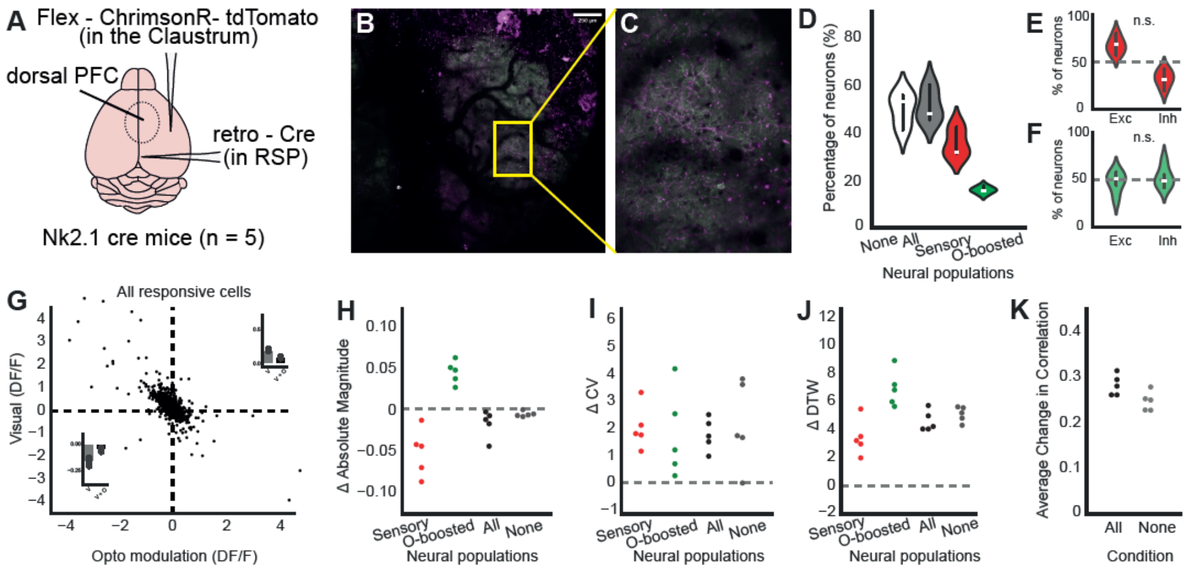

Supplementary Figure 5: Claustrum axon activation enhances neural flexibility in the inhibitory cells and enhance homogeneity in dorsal prefrontal cortex

A) Schematic of the experimental setup for two-photon imaging of dorsal inhibitory PFC neurons expressing GCaMP6s (n = 5) in Nk2.1 mice. B) Stitched two-photon image of the dorsal surface of the brain showing GCaMP-expressing neurons (green) and claustrum axons labelled with tdTomato (magenta) within the imaging field of view. In this experiment, we presented visual and visual+opto stimuli, but did not include opto-only stimuli, so that there was no opto responsive subpopulation. C) The yellow box in panel B highlights the region magnified in panel C, where individual neurons are clearly visible. (D) Violin plot showing the percentage of neurons categorized into different neural populations. (E-F) Violin plots comparing the percentage of excitatory (Exc) and inhibitory (Inh) neurons within the neural populations categorized as Sensory and Opto-boosted, respectively. The n.s. (not significant) label indicates no significant difference between the proportions of excitatory and inhibitory neurons. (G) Scatter plot illustrating the relationship between visual response (ΔF/F) and opto modulation (ΔF/F) for all responsive cells. The plot shows a strong negative correlation between visual and optogenetic modulation ($r = -0.521$, $p < 0.001$) (H) Dot plot showing the change in absolute magnitude (Δ Absolute Magnitude) of neural responses across different neural populations under visual+opto stimuli. Sensory ($p = 0.019$ and opto-boosted ($p < 0.001$, paired t-test) subpopulation showed significant modulation in different directions. (I) Dot plot showing the change in the coefficient of variation (Δ CV) across the same neural populations, indicating variability in response magnitude across trials (sensory: $p = 0.792$; opto-boosted: $p = 0.712$, paired t-test). (J) Dot plot showing the change in Dynamic Time Warping (Δ DTW), a measure of temporal variability, across the same neural populations (sensory: $p = 0.020$; opto-boosted: $p = 0.058$, paired t-test). (K) Dot plot showing the average change in correlation across all conditions (All, None), highlighting the overall effect of optogenetic modulation on neural network coordination in the dPFC. Overall, the responses of inhibitory cells were modulated with claustrum axon modulation in a manner similar to that of CamKII-expressing cells

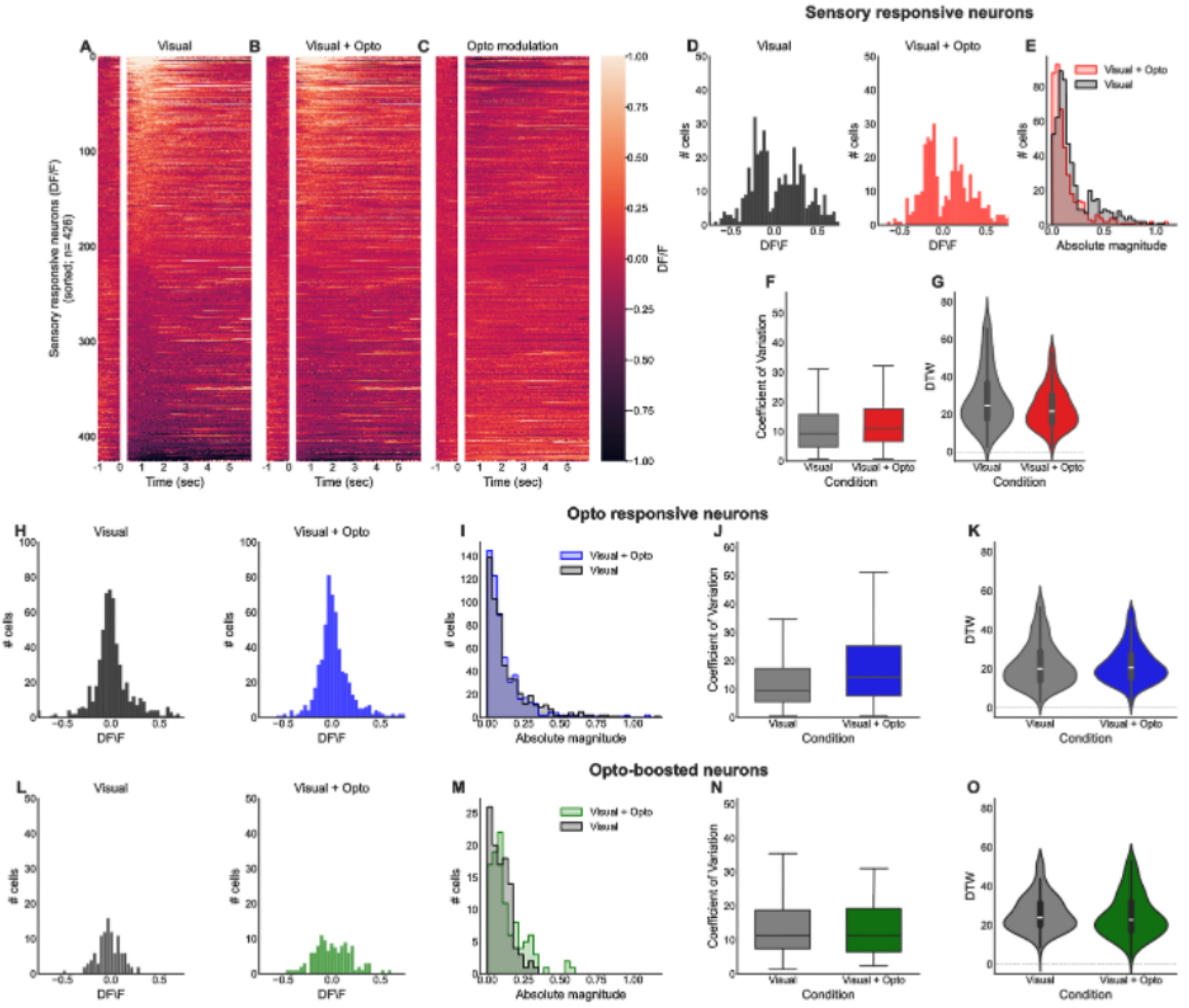

Supplementary Figure 6: **Optogenetic modulation of PFC responses during thalamus axon stimulation.**

As an additional control experiment, we expressed ChrimsonR in the thalamus and delivered widefield optogenetic stimulation to thalamic axons in the dorsal prefrontal cortex (dPFC), while recording calcium activity from CaMKII+ cells in dPFC using two-photon imaging. A total of 436 PFC neurons were recorded across 3 mice. Panels A–C show heatmaps of trial-averaged ΔF/F responses from sensory-responsive neurons in response to visual stimuli alone (A), visual plus optogenetic stimulation (B), and the resulting modulation (C; Visual+Opto – Visual). Panels D–F show histograms of ΔF/F responses (D), absolute response magnitude (E), and coefficient of variation (F). Panel G presents violin plots of dynamic time warping (DTW) distance between visual and visual+opto conditions. Panels H–K and L–O show the same analysis for cells classified as opto-responsive (H–K) and opto-boosted (L–O), respectively. This dataset provides an additional comparison of how PFC neurons respond to visual input and optogenetic activation of thalamic inputs, using the same analysis framework as in Figure 3. This control dataset demonstrates that the effects observed in the main experiments are not generalizable across all subcortical inputs, and are not artifacts of the stimulation or imaging methodology, but rather reflect the specific modulatory role of claustrum input to PFC.

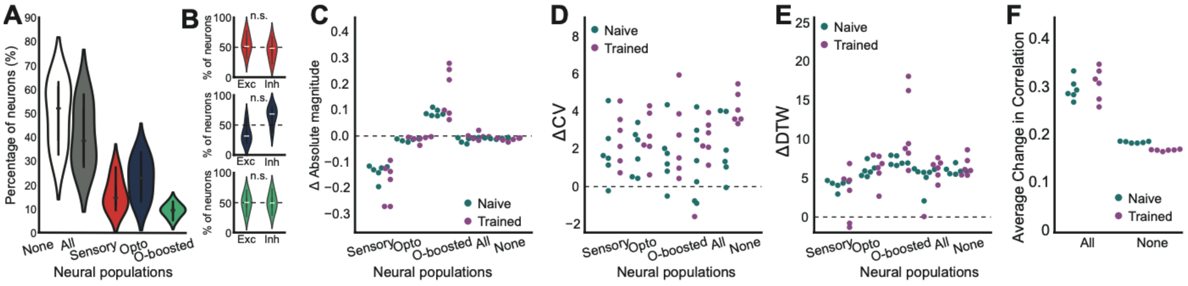

Supplementary Figure 7: Training did not change the claustrum axon silencing-induced neural flexibility and network homogeneity.

A) Violin plots displaying the percentage of responsive neurons across different neural populations: non-responsive (None), all responsive (All), sensory responsive, opto responsive, and opto-boosted neurons. B) Box plots comparing the percentages of excitatory (Exc) and inhibitory (Inh) responses within each neural population. C) Swarm plots for the absolute magnitude of neuronal responses across before (Naive) and after training (Trained). D) Same as C but for the coefficient of variation (ΔCV). E) Same as C but for dynamic time warping values (ΔDTW). E) The average change in cross-correlation coefficients for naive and trained data.

The fundamental mechanisms across subpopulations remained consistent after the training; the change in absolute magnitude (Fig. 5.1C, sensory p = 0.219; opto p = 0.438; opto-boosted p = 0.156, paired t-test), the change in the coefficient of variation (Fig. 5.1D, sensory p = 0.563; opto p = 0.438; opto-boosted p = 0.562, paired t-test), and the change in DTW (Fig. 5.1E, sensory p = 0.563; opto p = 0.844; opto-boosted p = 0.063, paired t-test) was greater than zero but did not differ in trained animals compared to naive animals across populations. The correlation analysis revealed no change in correlation among neuronal responses in trained animals compared to naive animals (Fig. 5.1F). A two-way ANOVA showed no significant main effects of training (F (1,20) = 0.112, p = 0.741 (responsiveness: F (1, 20) = 222.034, p < 0.001); interaction F (1, 20) = 2.753, p = 0.113).
