# Supplementary material for: The claustrum enhances neural variability by modulating the responsiveness of the prefrontal cortex
Source: Nat Commun. 2025 Nov 28;16:10767. doi: 10.1038/s41467-025-65809-6 (PMC12663370; doi:10.1038/s41467-025-65809-6)
Supplement: Supplementary file 2 — Reporting Summary [file 41467_2025_65809_MOESM2_ESM.pdf]

nature portfolio

Corresponding author(s): NCOMMS-25-19219-T

Last updated by author(s): Oct 6, 2025

Reporting Summary

Nature Portfolio wishes to improve the reproducibility of the work that we publish. This form provides structure for consistency and transparency in reporting. For further information on Nature Portfolio policies, see our Editorial Policies and the Editorial Policy Checklist.

Statistics

For all statistical analyses, confirm that the following items are present in the figure legend, table legend, main text, or Methods section.

n/a	Confirmed	
☐	☒	The exact sample size (*n*) for each experimental group/condition, given as a discrete number and unit of measurement
☐	☒	A statement on whether measurements were taken from distinct samples or whether the same sample was measured repeatedly
☐	☒	The statistical test(s) used AND whether they are one- or two-sided<br>*Only common tests should be described solely by name; describe more complex techniques in the Methods section.*
☐	☒	A description of all covariates tested
☐	☒	A description of any assumptions or corrections, such as tests of normality and adjustment for multiple comparisons
☐	☒	A full description of the statistical parameters including central tendency (e.g. means) or other basic estimates (e.g. regression coefficient) AND variation (e.g. standard deviation) or associated estimates of uncertainty (e.g. confidence intervals)
☐	☒	For null hypothesis testing, the test statistic (e.g. F, t, r) with confidence intervals, effect sizes, degrees of freedom and P value noted<br>*Give P values as exact values whenever suitable.*
☒	☐	For Bayesian analysis, information on the choice of priors and Markov chain Monte Carlo settings
☐	☒	For hierarchical and complex designs, identification of the appropriate level for tests and full reporting of outcomes
☐	☒	Estimates of effect sizes (e.g. Cohen's *d*, Pearson's *r*), indicating how they were calculated

Our web collection on statistics for biologists contains articles on many of the points above.

Software and code

Policy information about availability of computer code

Data collection	Behavioural training and photostimulation was controlled by custom written code in Python and C, available from the authors upon request. Online imaging analysis was done using STAMovieMaker (https://github.com/llerussell/STAMovieMaker) and custom code (will be available upon publication). Offline pre-processing imaging analysis was performed using Suite2p (Pachitariu et al., 2016) Software versions: pyControl: version 1.4 https://github.com/pyControl/code, Python: version 3.7 , 3.8 and 3.9 Matlab: version 2020b Blimp: https://github.com/Packer-Lab/blimp Naparm: https://github.com/llerussell/Naparm, Blink SDK: Meadowlark Optics version 1, PackIO : https://github.com/apacker83/PackIO, PrairieView: Bruker corporation version 5.5<br><br>Custom code for stimulus generation can be found: https://github.com/huriyeatg/clapfcstimulation/tree/main/stimuli_onePhotonStimulation
Data analysis	All data analysis and visualisation was performed in Python 3.9 using custom written code, which will be made publicly available on Github upon publication. Software versions: Python: version 3.9 Scipy: version 1.6 (package contains e.g. Scikit-learn: version 0.24), STAMovieMaker: https://github.com/llerussell/STAMovieMaker, Suite2p: version 0.9.<br><br>Main data analysis repository: https://github.com/huriyeatg/clapfcstimulation

For manuscripts utilizing custom algorithms or software that are central to the research but not yet described in published literature, software must be made available to editors and reviewers. We strongly encourage code deposition in a community repository (e.g. GitHub). See the Nature Portfolio guidelines for submitting code & software for further information.

Data

Policy information about availability of data

All manuscripts must include a data availability statement. This statement should provide the following information, where applicable:

- Accession codes, unique identifiers, or web links for publicly available datasets
- A description of any restrictions on data availability
- For clinical datasets or third party data, please ensure that the statement adheres to our policy

The functional two-photon calcium imaging recordings that are presented in this manuscript is available : https://doi.org/10.6084/m9.figshare.30164857.v2

Research involving human participants, their data, or biological material

Policy information about studies with human participants or human data. See also policy information about sex, gender (identity/presentation), and sexual orientation and race, ethnicity and racism.

Reporting on sex and gender	N/A
Reporting on race, ethnicity, or other socially relevant groupings	N/A
Population characteristics	N/A
Recruitment	N/A
Ethics oversight	N/A

Note that full information on the approval of the study protocol must also be provided in the manuscript.

Field-specific reporting

Please select the one below that is the best fit for your research. If you are not sure, read the appropriate sections before making your selection.

☒ Life sciences ☐ Behavioural & social sciences ☐ Ecological, evolutionary & environmental sciences

For a reference copy of the document with all sections, see nature.com/documents/nr-reporting-summary-flat.pdf

Life sciences study design

All studies must disclose on these points even when the disclosure is negative.

Sample size	Sample sizes were chosen based on previous studies (e.g. Dalgleish et al. 2020) that showed statistical inferences can be drawn, in studies using two-photon calcium imaging & two- photon optogenetics, with N ≈ 10 mice. We did not exceed this number to adhere to the 3Rs (specifically reduction).
Data exclusions	In passive imaging during visual and photostimulation fixed number of repeated trials were used- no data was excluded. During the 250ms of photostimulation, the frames occuring while the photostimulation laser was on was excluded from the analysis due to artifactual crosstalk in the imaging channel. (see Methods)<br>In behavioural training, performance of sessions without imaging was not reported and 2 mice who failed to learn the stimulus-action association was excluded. (see Methods).
Replication	To verify reproducibility of our experimental findings, we:<br>- performed all experiments N=11 times using 6 mice<br>- performed all data analysis on all these N=11 data sets, and always report all main results (either all N=11, or by presenting the relevant statistics).
Randomization	Randomisation across trial types for each recording session was used was used.
Blinding	Blinding was not applicable to this study as we did not divide our test subjects into groups that could be subject to selection bias or other confounds.

Reporting for specific materials, systems and methods

We require information from authors about some types of materials, experimental systems and methods used in many studies. Here, indicate whether each material, system or method listed is relevant to your study. If you are not sure if a list item applies to your research, read the appropriate section before selecting a response.

Materials & experimental systems

n/a	Involved in the study
☒	☐ Antibodies
☒	☐ Eukaryotic cell lines
☒	☐ Palaeontology and archaeology
☐	☒ Animals and other organisms
☒	☐ Clinical data
☒	☐ Dual use research of concern
☒	☐ Plants

Methods

n/a	Involved in the study
☒	☐ ChIP-seq
☒	☐ Flow cytometry
☒	☐ MRI-based neuroimaging

Animals and other research organisms

Policy information about studies involving animals; ARRIVE guidelines recommended for reporting animal research, and Sex and Gender in Research

Laboratory animals	Male and female C57BL/6J or Nkx2.1Cre;Ai9 background mice were used in these experiments. Twenty four GCaMP6s transgenic mice (CamKIIa-tTa x B6;DBA-Tg(tetO-GCaMP6s)2Niell/J) were used for imaging of the claustrum axon activation and inhibition experiments. Additionally, five Nkx2.1-Cre x tdTomato were used for inhibitory neuron imaging experiments. Mice were between 5-11 weeks of age when surgery was performed. Mice were housed at room temperature (20–22 °C) on a standard light-dark cycle and humidity of ~40%.
Wild animals	No wild animals were used in the study.
Reporting on sex	Male as well as female animals were used in the study.
Field-collected samples	No field-collected samples were used in the study.
Ethics oversight	All experimental procedures involving animals were conducted in accordance with the UK animals in Scientific Procedures Act (1986).

Note that full information on the approval of the study protocol must also be provided in the manuscript.

Plants

Seed stocks	N/A
Novel plant genotypes	N/A
Authentication	N/A
